# Haplotype graph analysis of *PdR1* uncovers resistance diversity to Pierce's disease in *Vitis arizonica* and its hybrids

**DOI:** 10.1093/g3journal/jkag115

**Published:** 2026-04-30

**Authors:** Mélanie Massonnet, Mirella Zaccheo, Noé Cochetel, Rosa Figueroa-Balderas, Summaira Riaz, Dario Cantu

**Affiliations:** Department of Viticulture and Enology, University of California Davis, Davis, CA 95616, United States; Department of Viticulture and Enology, University of California Davis, Davis, CA 95616, United States; Department of Viticulture and Enology, University of California Davis, Davis, CA 95616, United States; Department of Viticulture and Enology, University of California Davis, Davis, CA 95616, United States; U.S. Department of Agriculture-Agricultural Research Service, San Joaquin Valley Agricultural Center, Parlier, CA 93648, United States; Department of Viticulture and Enology, University of California Davis, Davis, CA 95616, United States; Genome Center, University of California Davis, Davis, CA 95616, United States

**Keywords:** *Vitis arizonica*, Pierce's disease, *Xylella fastidiosa*, disease resistance, haplotype-resolved genomes, sequence graph

## Abstract

Previous genetic mapping studies indicate that multiple haplotypes of the *Pierce's disease (PD) Resistance 1 (PdR1)* locus occur in *Vitis arizonica* and its hybrids. To characterize sequence diversity at this locus, we assembled chromosome-scale diploid genomes for four PD-resistant (PD-R) accessions: b43-17 (*PdR1a^+^/PdR1b^+^*), the backcross 07744-094 (*PdR1c^+^/PdR1^−^*), b46-43 (*PdR1e^+^/PdR1f^+^*), and b42-26 (*PdR1^−^/PdR1^−^*), which displays quantitative PD resistance not associated with *PdR1*. Haplotype resolution of *PdR1a*, *PdR1b*, *PdR1c*, and *PdR1e* revealed substantial variation in intergenic repeat content and gene composition between *PdR1* haplotypes and their alternatives at the *PdR1* locus not associated with PD resistance phenotype (*PdR1*^−^), as well as among *PdR1* haplotypes, demonstrating extensive sequence diversity at the *PdR1* locus. Sequence graph analysis uncovered substantial structural divergence concentrated in approximately one quarter of the locus, together with smaller-scale variation across haplotypes. This analysis identified *PdR1*-specific graph nodes, showing that *PdR1a* and *PdR1b* share most of their *PdR1*-specific features, whereas *PdR1c* contains the highest number of private nodes, followed by *PdR1e*. Integration of sequence graph features with gene expression data further refined a set of defense-related candidate genes within *PdR1c*. Together, these results identify candidate genes for functional validation and indicate that multiple resistance determinants co-localized within the *PdR1* locus may contribute to PD resistance, highlighting opportunities for targeted genetic improvement strategies.

## Teaser

Pierce’s disease is a major threat to grape production, and resistance has been traced to a genomic region called *PdR1* in wild grape relatives. Massonnet *et al*. generated high-quality genomes for grape accessions carrying different *PdR1* variants and compared their DNA sequences. The authors found that *PdR1* shows extensive structural and gene-level variation, including multiple resistance-associated genes within the same locus. By combining genome comparisons with gene expression data, they identified candidate genes likely involved in resistance. These results provide new targets for functional testing and practical genomic information that can support the development of disease-resistant grape varieties.

## Introduction

Pierce's disease (PD) is caused by the bacterium *Xylella fastidiosa* ssp. *fastidiosa* (hereafter *Xf*), which colonizes grapevine xylem. In California, PD remained limited until the late 1990s when the introduction of the non-native glassy-winged sharpshooter (*Homalodisca vitripennis*) in the southern part of the state led to a rapid rise in disease incidence (Rapicavoli et al. 2018). These insects, along with the native blue-green sharpshooter (*Graphocephala atropunctata*) and **s**pittlebug species (family Cercopidae), are principal vectors of *Xf.* All *Vitis vinifera* cultivars are susceptible to PD, whereas some wild grape species from the southern US and northern Mexico, including *V. arizonica* and its hybrids, are PD-resistant.

In grapes, *Xf* colonization triggers the formation of balloon-shaped tyloses in the xylem, which help block pathogen spread and limit air embolisms (Castro et al. 2021). While this defense response is effective in PD-resistant grapes, tylose formation is spatially and temporally misaligned with *Xf* colonization in PD-susceptible cultivars, leading to extensive vessel occlusion (Sun et al. 2013). Coupled with systemic *Xf* spread through bacterial biofilms, excess tylose development sharply reduces stem hydraulic conductivity in susceptible grapevines (Rapicavoli et al. 2018). This results in leaf scorching, berry desiccation, irregular periderm development, abnormal leaf abscission, and vine death (Rapicavoli et al. 2018).

PD management mostly relies on the use of insecticides to control vectors and the removal of infected plants (Haviland et al. 2021). Despite these practices, annual production loss is estimated at $56.1 million per year in California (Tumber et al. 2014). In addition, growing application restrictions and emerging pesticide resistance have made this approach unsustainable (Haviland et al. 2021). Therefore, the long-term solution is the development of grape cultivars with strong and durable PD resistance combined with desirable agronomic and enological traits. Such resistance can be achieved through the introgression of functionally diverse PD resistance-associated genes or alleles into *V. vinifera* (Michelmore et al. 2013).

One resistance (*R*) locus located on chromosome 14, *PD Resistance 1* (*PdR1*), was identified in grapes through genetic mapping (Krivanek et al. 2006; Riaz et al. 2006). Using marker-based maps and tightly linked molecular markers, putative *PdR1* haplotypes were distinguished among *V. arizonica* accessions and their hybrids based on marker size polymorphisms rather than sequence data (Riaz et al. 2008, 2018). The presence of multiple marker-defined haplotypes at the same locus (*PdR1a-f*) suggests that PD resistance may involve different genes or allelic variants within *PdR1* (Table 1). The diploid genome of *V. arizonica* b40-14, which carries *PdR1c* and *d* (*PdR1c*^+^*/PdR1d*^+^), together with a genome-wide association and gene expression profiling, enabled the identification of four candidate *R* genes encoding extracellular receptors: two leucine-rich repeat (LRR) receptor-like proteins (RLPs), one LRR receptor-like kinase (LRR-RLK), and one lysin motif (LysM)-RLK (Morales-Cruz et al. 2023). However, limited haplotype information prevented confirmation of the locus phasing and whether the studied *PdR1* haplotype corresponded to the *c* or *d* haplotype of b40-14.

**Table 1.** List of the PD-resistant grape accessions whose genome was sequenced in this study.

Accession	*PdR1* composition	Species	Reference
b43-17	*PdR1a* ^+^ */PdR1b* ^+^	*V. arizonica × V. candicans* hybrid	Krivanek et al. 2006
			Riaz et al. 2008
07744-094	*PdR1c* ^+^/*PdR1*^−^	(*V. rupestris* Wichita refuge *× V. arizonica glabrous* b40-14) *× V. vinifera* cv. Airen	Riaz et al. 2018
b46-43	*PdR1e* ^+^ */PdR1f* ^+^	*V. arizonica glabrous × V. monticola* hybrid	Riaz et al. 2018
b42-26	*PdR1^−^/PdR1^−^*	*V. arizonica × V. girdiana* hybrid	Riaz et al. 2018

*PdR1b* has already been deployed in the University of California Davis grape breeding program and underlies the five PD-resistant cultivars released in 2019 (https://fps.ucdavis.edu/newsarticle.cfm?newsid=57). These cultivars have generated significant industry interest, motivating efforts to characterize additional *PdR1* haplotypes that could further strengthen resistance and improve its durability in future breeding. In this study, to better understand the range of resistance encoded at this locus, we assess sequence diversity among five *PdR1* haplotypes and its impact on genes potentially involved in *Xf* resistance. We sequenced, assembled, and annotated diploid chromosome-scale genomes for four PD-resistant accessions: b43-17 (*PdR1a*^+^*/PdR1b*^+^), the backcross 07744-094 (*PdR1c*^+^/*PdR1*^−^), b46-43 (*PdR1e*^+^*/PdR1f*^+^), and b42-26 (*PdR1*^−^/*PdR1*^−^), which displays quantitative PD resistance but not associated with *PdR1* (Table 1) (Riaz et al. 2018). After localizing the *PdR1* haplotypes using marker data and phasing them, we compared the gene content potentially associated with *Xf* resistance across five *PdR1* haplotypes and three alternative haplotypes. *PdR1*^−^ will be used to describe the alternative haplotype at the *PdR1* locus not associated with PD-resistance phenotype. A sequence graph built from both *PdR1* and *PdR1*^−^ haplotypes allowed us to identify variants specific to the *PdR1* haplotypes, compare these variants across haplotypes, and determine which ones altered the coding sequences of defense-related genes. Finally, we show how the information of the sequence graph, coupled with gene expression, can help to narrow down candidate genes among the refined region of *PdR1c* (Morales-Cruz et al. 2023).

## Materials and methods

### Plant material

Young leaves from the accessions b43-17 (*PdR1a*^+^*/PdR1b*^+^), 07744-094 (*PdR1c*^+^/*PdR1*^−^), b46-43 (*PdR1e*^+^*/PdR1f*^+^), and b42-26 (*PdR1*^−^/*PdR1*^−^) were collected, immediately flash frozen, and ground in liquid nitrogen. All four accessions were field-grown plants maintained at the Department of Viticulture and Enology, University of California, Davis, CA, at the time of collection. Accessions b43-17, 07744-094, b46-43, and b42-26 were planted in 2003, 2008, 2014, and 2015, respectively.

### DNA extraction, library preparation, and sequencing

High-molecular-weight genomic DNA was isolated from the young leaves of the four accessions like in Chin *et al*. (Chin et al. 2016). DNA concentration was evaluated using the DNA High Sensitivity Kit on a Qubit 2.0 Fluorometer (Life Technologies, CA, USA), and DNA purity with a Nanodrop 2000 spectrophotometer (Thermo Scientific, IL, USA). Assessment of the DNA integrity was done using the FEMTO Pulse system (Agilent, CA, USA). Preparation of the HiFi libraries was performed using the SMRTbell Express Template Prep Kit 2.0 (Pacific Biosciences, CA, USA) following the manufacturer's protocol. Libraries were size-selected using 3.1 X v/v of 35% Ampure PB beads (Pacific Biosciences, CA, USA), and sequenced on a PacBio Sequel II platform (DNA Technology Core Facility, University of California, Davis, CA, USA). Sequencing resulted in 54, 54, 57, and 40X of coverage of a 500-Mbp haplotype for b43-17 (*PdR1a*^+^*/PdR1b*^+^), 07744-094 (*PdR1c*^+^/*PdR1*^−^), b46-43 (*PdR1e*^+^*/PdR1f*^+^), and b42-26 (*PdR1*^−^/*PdR1*^−^), respectively (Supplementary Table 1).

### Genome assembly

HiFi reads were assembled using hifiasm (Cheng et al. 2021) with the option “-n 13” for b43-17 (*PdR1a*^+^*/PdR1b*^+^), 07744-094 (*PdR1c*^+^/*PdR1*^−^), and b42-26 (*PdR1*^−^/*PdR1*^−^), and “-n 25” for b46-43 (*PdR1e*^+^*/PdR1f*^+^) (Supplementary Table 2). Diploid chromosome-scale pseudomolecules were scaffolded from contigs using HaploSync v1.0 (Minio et al. 2022) and a consensus grape synteny map (Cochetel et al. 2025) (Supplementary Table 3). Telomere repeat units were searched as described in Massonnet *et al*. (Massonnet et al. 2025) using TIDK v.0.2.63 (https://github.com/tolkit/telomeric-identifier). Gene space completeness of the genome assemblies was evaluated using BUSCO v.5.4.7 with the library embryophyte_odb10 (Waterhouse et al. 2018).

### Genome annotation

Repeat and gene annotations were performed as in Massonnet *et al*. (Massonnet et al. 2025). Repeat annotation was made using RepeatMasker v.4.1.7-p1 (Smit et al. 2013) and a grape repeat library (Minio et al. 2019). PN40024 V5.1 predicted protein-coding gene sequences (https://grapedia.org/t2t_annotation/), along with the high-quality transcripts obtained from the Iso-Seq full-length reads of *V. arizonica* b40-14 (Cochetel et al. 2023) and *V. vinifera* cv. Cabernet Sauvignon (Minio et al. 2019), were used as transcriptomic evidence for the gene annotation. For each accession, a set of high-quality gene models was created using the PASApipeline v2.5.3 (https://github.com/PASApipeline/PASApipeline), GMAP v.2024-11-20 (Wu and Watanabe 2005), pblat v.2.5.1 (Wang and Kong 2019), and the transcriptomic evidence aforementioned. The set of high-quality gene models was then used as input for the *ab initio* predictors Augustus v.3.5.0 (Stanke et al. 2006) and GeneMark v.3.68_lic (Lomsadze et al. 2005). Consensus models from the gene models produced by the *ab initio* predictions and PASA were made using EvidenceModeler v.2.1.0 (Haas et al. 2008). Gene models encoding protein sequences with a length less than 50 amino acids, or in-frame stop codons, or lacking a start methionine or a stop codon, were removed.

### Functional annotation

Predicted proteins of the four genomes were aligned onto the proteins of *Arabidopsis thaliana* (Araport11_genes.201606.pep.fasta; https://www.arabidopsis.org/download/index.jsp) using BLASTP v.2.12.0+ (Camacho et al. 2009). Homologous proteins were identified by: first, keeping the alignments with an identity greater than 30%, and both reference:query and query:reference coverages between 75 and 125%; second, selecting the alignment with the highest product of identity by query coverage by reference coverage for each grape protein. The same method was used to determine homologous proteins in *V. vinifera* PN40024 genome V1 annotation and V5.1 annotations (Jaillon et al. 2007; Shi et al. 2023).

To identify protein domains, hmmsearch from HMMER v.3.3.1 (Eddy 2011) was used with the Pfam-A Hidden Markov Models (HMM) database (El-Gebali et al. 2019) (downloaded on March 12th, 2025). Protein domains with an independent E-value less than 1.0, and at least 50% of the HMM, were selected (Supplementary Tables 4–7).

Transmembrane helices were predicted with TMHMM2 v2.0c (Krogh et al. 2001).

### *PdR1* region localization

The five *PdR1* haplotypes and the three *PdR1*^−^ haplotypes were located by *in silico* amplification of two *PdR1*-associated markers, VMCNg2b7.2 and UDV095 (Riaz et al. 2008), on the genome assemblies using dispr (https://github.com/douglasgscofield/dispr).

### Haplotyping

To identify *PdR1c* in 07744-094 (*PdR1c*^+^/*PdR1*^−^) and *PdR1b* in b43-17 (*PdR1a*^+^*/PdR1b*^+^), we used short DNA-seq reads from *V. arizonica* b40-14 (*PdR1c*^+^/*PdR1d*^+^) and five backcrossed cultivars carrying *PdR1b*: Ambulo blanc, Caminante blanc, Camminare noir, Errante noir, and Paseante noir (Morales-Cruz et al. 2023), and a coverage analysis of unambiguous alignments. Adapter sequence removal and quality read selection was performed using Trimmomatic v.0.36 (Bolger et al. 2014) with the following parameters: “LEADING:3 TRAILING:3 SLIDINGWINDOW:10:20 MINLEN:36 CROP:150”. High-quality reads of *V. arizonica* b40-14 and the five backcrossed cultivars were aligned on 07744-094 and b43-17 genomes, respectively, using BWA v0.7.17 (Li and Durbin 2009) with the parameter “-a” to report all multiple alignments. Unambiguous alignments (i.e. with no mismatches) were retained with bamtools filter v.2.5.1 (Barnett et al. 2011) and the option “NM:0”. Base coverage was obtained using genomecov from BEDTools v2.29.1 (Quinlan 2014). Coverage in repetitive elements was removed using BEDTools intersect. Median coverage per window of 10-kbp was computed by BEDTools map. Median coverage per 10-kbp window was normalized by dividing by the sequencing coverage of the accessions. SNPs and INDELs between the two haplotypes of b43-17 were called using NUCmer from MUMmer v.4.0.0 (Marçais et al. 2018) with the “--mum” option and show-snps with the parameters “-Clr”.

### Sequence graph construction

Sequence graph of the five *PdR1* haplotypes and the three *PdR1*^−^ alternative haplotypes was built using the nf-core/pangenome pipeline (Heumos et al. 2024) with NextFlow v.25.04.6 (Di Tommaso et al. 2017) and the parameters “-r 1.1.2 -profile singularity”. 2D representation was generated using ODGI v0.9.3 (Guarracino et al. 2022).

### Alignment of PD resistance-associated kmers

PD resistance-associated kmers from *V. arizonica* were retrieved from Morales-Cruz *et al*. (Morales-Cruz et al. 2023). They were mapped on the genome of 07744-094 (*PdR1c*^+^/*PdR1*^−^) using BLASTN v.2.12.0+ (Camacho et al. 2009) and a word size of 8 bp. Full-length and perfect alignments (i.e. 100% coverage and identity) were retained.

### Gene expression analysis

RNA-seq reads of three PD-resistant 07744 lines were retrieved from NCBI BioProject PRJNA956994 (Morales-Cruz et al. 2023). High-quality reads were obtained using Trimmomatic v.0.36 (Bolger et al. 2014) with the following parameters: “LEADING:3 TRAILING:3 SLIDINGWINDOW:10:20 MINLEN:36” (Supplementary Table 11). Transcript abundance was assessed with Salmon v.1.5.1 (Patro et al. 2017). A transcriptome index file was built using the protein-coding sequences of 07744-094 and its genome as decoy, using the parameters “-k 31 --keepDuplicates”. Transcript abundance was quantified with the parameters “--gcBias --seqBias --validateMappings”. Gene expression in transcript per million (TPM) was extracted from quantification files with the R package tximport v.1.30.0 (Soneson et al. 2016). Differential gene expression analysis was performed using DESeq2 v.1.42.1 (Love et al. 2014).

## Results

### Diploid chromosome-scale genome assemblies

To characterize the sequence diversity among the *PdR1* haplotypes, we sequenced the genomes of three PD-resistant *V. arizonica* hybrids: *V. arizonica* x *V. candicans* hybrid b43-17 (*PdR1a*^+^*/PdR1b*^+^), *V. arizonica glabrous* x *V. monticola* hybrid b46-43 (*PdR1e*^+^*/PdR1f*^+^), and *V. arizonica* x *V. girdiana* hybrid b42-26 (*PdR1*^−^/*PdR1*^−^) which has quantitative PD resistance not associated with *PdR1* (Table 1) (Riaz et al. 2018), using long-read DNA sequencing (PacBio HiFi reads; Supplementary Table 1). In addition, we sequenced the genome of the backcross line 07744-094 to obtain the *PdR1c* haplotype from *V arizonica* b40-14 (*PdR1c*^+^/*PdR1d*^+^), because in the available b40-14 genome, the two *PdR1* haplotypes are fragmented and not haplotype-phased (Supplementary Fig. 1) (Morales-Cruz et al. 2023).

Draft diploid genomes were composed of an average of 192 ± 43.4 contigs (Supplementary Table 2). After pseudomolecule reconstruction, each set of 19 chromosomes spanned 491.9 ± 67.8 Mbp, with unanchored contigs accounting for only 1.7 ± 0.8% of the assembly (16.7 ± 8.1 Mbp) (Table 2; Supplementary Table 3). On average, 99.2 ± 0.2% of expected single-copy orthologs (BUSCOs) were detected in each set of 19 chromosomes, indicating a high completeness. Furthermore, we detected telomeric repeats at both ends of 30, 28, 24, and 16 chromosomes for 07744-094, b42-26, b46-43, and b43-17, respectively, and at one end of 8, 10, 11, and 21 chromosomes, respectively, out of 38. These results indicate that the four haplotype-resolved assemblies are highly contiguous and complete.

**Table 2.** Statistics of the four genome assemblies.

Accession	b43-17			07744-094			b46-43			b42-26		
*PdR1* composition	*PdR1a* ^+^/*PdR1b*^+^			*PdR1c* ^+^/*PdR1*^−^			*PdR1e* ^+^/*PdR1f*^+^			*PdR1* ^−^/*PdR1*^−^		
Haplotype	Hap1	Hap2	Unp	Hap1	Hap2	Unp	Hap1	Hap2	Unp	Hap1	Hap2	Unp
Assembly length (Mbp)	502.1	488.3	15.4	484.6	491.8	13.7	501.1	489.8	9.4	493.6	484.1	28.1
Number of sequences	19	19	81	19	19	70	19	19	56	19	19	93
Telomeres	29	24	0	33	35	0	29	30	0	33	33	0
Number of genes (x1,000)	39.2	38.3	1.0	37.5	36.1	3.3	32.1	31.4	0.8	33.1	32.4	2.1
Repeats (%)	56.3	56.0	79.9	56.5	56.0	76.8	57.3	56.6	69.7	56.1	55.8	78.9
BUSCOs (%)	99.2	98.9	1.1	99.4	99.5	0.4	99.1	99.1	0.1	99.1	99.1	1.8

Abbreviations: Hap, Haplotype; Unp, Unplaced contigs.

### *PdR1* localization and haplotyping

The boundaries of the *PdR1* and *PdR1*^−^ haplotypes were located through *in silico* amplification of the *PdR1*-linked markers VMCNg2b7.2 and UDV095 (Riaz et al. 2008) (Supplementary Table 8). Three additional markers (Ch14-70, Ch14-77, and Ch14-02) were used to confirm their localization (Riaz et al. 2018). Amplification revealed amplicon-size variation between accessions for all markers. Variation within accessions, reflecting differences between the two homologous chromosomes, was also observed for at least one marker in each accession, except b46-43, whose two haplotypes produced identical amplicon sizes for all markers (Supplementary Table 8). The size of the *PdR1* region varied across the four grape accessions, providing further evidence of haplotype diversity (Table 3; Supplementary Table 9). Substantial differences in locus size between haplotypes were found in 07744-094 (*PdR1c*^+^/*PdR1*^−^) (1,103.9 and 842.8 kbp for Haplotype 1 and 2, respectively) and b42-26 (*PdR1*^−^/*PdR1*^−^) (969.7 and 848.1 kbp on Haplotype 1 and 2, respectively). In contrast, the two *PdR1* haplotypes of b43-17 (*PdR1a*^+^*/PdR1b*^+^) differed by only 1.7 kbp (889.4 and 887.7 kbp on Haplotype 1 and 2, respectively), and the two haplotypes of b46-43 (*PdR1e*^+^*/PdR1f*^+^) were identical except for a single SNP located in a repetitive region at 472,149 bp after VMCNg2b7.2. Because our aim is to investigate the sequence diversity among *PdR1* haplotypes, only Haplotype 1 of b46-43 was retained for the subsequent analyses and named *PdR1e*.

**Table 3.** Features of the *PdR1* and *PdR1*^−^ haplotypes.

Accession	b43-17		07744-094		b46-43	b42-26	
*PdR1* composition	*PdR1a* ^+^/*PdR1b*^+^		*PdR1c* ^+^/*PdR1*^−^		*PdR1e* ^+^/*PdR1f*^+^	*PdR1* ^−^/*PdR1*^−^	
Haplotype	Hap1 *PdR1b*	Hap2 *PdR1a*	Hap1 *PdR1*^−^	Hap2 *PdR1c*	Hap1 *PdR1e*	Hap1 *PdR1*^−^	Hap2 *PdR1*^−^
Length (kbp)	889.4	887.7	1,103.9	842.8	877.5	969.7	848.1
Intergenic repeat (kbp)	303.4	304.0	452.4	291.4	282.4	371.3	318.5
Gene loci	118	118	125	110	96	134	115
Defense-related genes	42	42	47	40	38	45	40
LRR-RLP	18	18	24	16	15	21	17
Peroxidase	1	1	1	2	1	1	1
MPL1-like protein	2	2	1	1	2	2	1
Calcium-binding EF-hand family protein gene	1	1	1	1	0	1	1

Defense-related genes with different numbers across the haplotypes are depicted in this table. Number of defense-related genes composing each haplotype can be retrieved in Supplementary Table 11. Gene content of each haplotype and their defense-related category and subcategory is detailed in Supplementary Tables 12–18. Abbreviations: Hap, Haplotype; LRR-RLP, leucine-rich repeat receptor-like proteins; MPL1, *Myzus persicae*-induced lipase 1

**Fig. 1.**
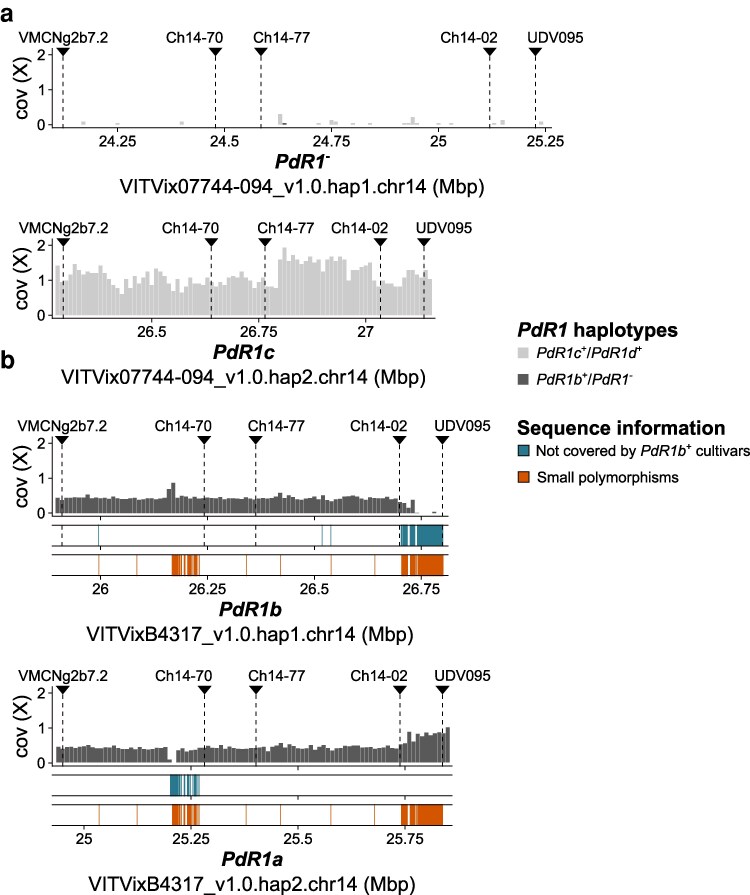
*PdR1* haplotyping of 07744-094 (*PdR1c*^+^/*PdR1*^−^) and b43-17 (*PdR1a*^+^*/PdR1b*^+^). a) Normalized median DNA-seq coverage per 10 kbp of b40-14 (*PdR1c*^+^/*PdR1d*^+^) on the diploid genome of 07744-094 (*PdR1c*^+^/*PdR1*^−^). b) Average of the normalized median DNA-seq coverage per 10 kbp of five *PdR1b*^+^ cultivars on the genome of b43-17 (*PdR1a*^+^*/PdR1b*^+^). Bases not covered by any *PdR1b*^+^ cultivar, and small polymorphisms between the two haplotypes of b43-17 (*PdR1a*^+^*/PdR1b*^+^) are depicted for each haplotype. Chromosomal position of the *PdR1*-associated genetic markers is indicated by black triangles and dashed lines.

Because 07744-094 (*PdR1c*^+^/*PdR1*^−^) is a backcross to *V. vinifera*, one haplotype derives from *V. arizonica* (carrying *PdR1c*) and the other from *V. vinifera* (*PdR1*^−^). Accordingly, we used short DNA-seq reads of b40-14 (*PdR1c*^+^/*PdR1d*^+^) to distinguish *PdR1c* from its homologous *V. vinifera PdR1*^−^ haplotype. Normalized median base coverage (per 10 kbp) was evaluated on both haplotypes of 07744-094 using only reads that aligned perfectly (i.e., without any mismatch). This strict criterion was applied to prevent calculating base coverage from reads carrying SNPs. Short DNA-seq reads of b40-14 covered the entire region at 1.1 ± 0.3× on the Haplotype 2 of chromosome 14, whereas little to no coverage was observed on Haplotype 1 (Fig. 1a). This indicates that *PdR1c* is located on Haplotype 2 of 07744-094 genome, while Haplotype 1 corresponds to the *V. vinifera PdR1*^−^ haplotype. Interestingly, coverage on Haplotype 2 between markers Ch14-77 and Ch14-02 (26.8—27.01 Mbp) averaged 1.5 ± 0.2×, suggesting that this region is quite homozygous between *PdR1c* and *PdR1d*.

Conversely, we assessed the normalized median base coverage of five PD-resistant BC_4_ and BC_5_ cultivars carrying only *PdR1b* (Morales-Cruz et al. 2023) to distinguish *PdR1b* from *PdR1a* in b43-17 (Fig. 1b; Supplementary Figs. 2 and 3). The average coverage across the five *PdR1b*^+^ cultivars was 0.4 ± 0.1× between markers VMCNg2b7.2 and Ch14-02 on both haplotypes of the b43-17 genome, suggesting that the region is quite homozygous in *PdR1a* and *PdR1b*. In the 3' portion of the locus, between the markers Ch14-02 and UDV095, Haplotype 1 showed little to no coverage, whereas 0.7 ± 0.1× coverage was found on Haplotype 2. This suggests that the five PD-resistant cultivars likely possess this part of the locus. In addition, a short region between markers VMCNg2b7.2 and Ch14-70 (from 25.2 to 25.21 Mbp) was uncovered in Haplotype 2, while a coverage peak (∼0.8×) was observed at 26.17 Mbp on Haplotype 1. The localized differences in coverage between haplotypes could reflect either recombination or assembly-related haplotype switches. Remarkably, small polymorphisms (< 50 bp) between the two haplotypes of b43-17 were mostly located in the same two regions (Fig. 1b). Furthermore, no sign of haplotype switch in these two regions could be observed on the alignment of the HiFi reads of b43-17 on the genome (Supplementary Figs. 4 and 5). Altogether, these results indicate that a recombination event occurred in the 3′ of the locus during the breeding of the five PD-resistant cultivars, and that *PdR1a* and *PdR1b* are located on Haplotype 2 and Haplotype 1 of chromosome 14 of b43-17, respectively.

### *PdR1* repeat and gene content

To determine the origin of *PdR1* haplotype size variation, we annotated the repeat and gene content of the four genomes. Annotation of repetitive elements showed that the total length of intergenic repeats in *PdR1* haplotypes ranged from 452.4 kb in the *V. vinifera PdR1*^−^ to 303.4 kb in *PdR1b* of b43-17 (Table 3, Supplementary Table 10). These data indicate that *PdR1* haplotype size differences are primarily driven by variation in intergenic repeat content.

The number of annotated gene loci ranged from 96 in *PdR1e* to 134 in the *PdR1*^−^ of b42-26 Haplotype 1. Based on protein domain composition and homology to *A. thaliana* proteins, we identified genes potentially involved in plant responses to biotic stress in each haplotype (Supplementary Tables 11–18). Defense-related genes within *PdR1* haplotypes included those encoding extracellular receptors and transcription factors, as well as genes involved in reactive oxygen species (ROS) production and scavenging, cell wall modification, programmed cell death, ethylene- and jasmonic acid–mediated signaling, and genes with putative roles in biotic stress responses of unknown mechanism (Supplementary Table 11). The number of defense-related genes ranged from 38 in *PdR1e* to 47 in the *V. vinifera PdR1*^−^ (Table 3; Supplementary Table 11).

Differences in defense-related gene content among *PdR1* haplotypes were mainly driven by variation in genes encoding LRR-RLPs between markers Ch14-77 and Ch14-02, which ranged from 16 to 24 genes. In addition, *PdR1c* contained two peroxidase-coding genes, compared with a single gene in the other haplotypes. Two genes encoding homologs of *Myzus persicae*–induced lipase 1 (MPL1) from *A. thaliana* were present in *PdR1a*, *PdR1b*, *PdR1e*, and the *PdR1*^−^ Haplotype 1 of b42-26, whereas only one *MPL1* homolog was detected in the remaining haplotypes. In *A. thaliana*, MPL1 restricts *Fusarium graminearum* infection by limiting jasmonic acid accumulation (Alam et al. 2022). A gene encoding a calcium-binding EF-hand family protein was present in all haplotypes except *PdR1e*.

Altogether, variation in intergenic repeat and gene composition, particularly among defense-related genes, provides clear evidence of sequence diversity between *PdR1* and *PdR1^−^* haplotypes, as well as among the *PdR1* haplotypes themselves.

### *PdR1* sequence graph

**Fig. 2.**
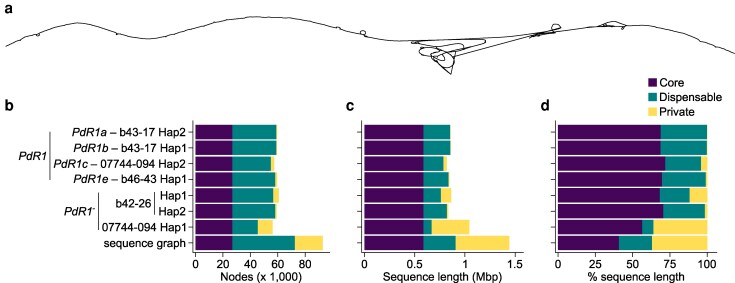
Sequence graph of the *PdR1* and *PdR1*^−^ haplotypes. a) 2D visualization of the sequence graph built using four *PdR1* haplotypes: *PdR1a* and *PdR1b* of b43-17, *PdR1c* from 07744-094 (*PdR1c*^+^/*PdR1*^−^), *PdR1e* of b46-43, and three *PdR1*^−^ haplotypes: the two haplotypes of b42-26 (*PdR1*^−^/*PdR1*^−^), and the *V. vinifera* haplotype of 07744-094. Number of nodes (b), cumulative sequence length (c), and percentage of sequence length (d), made of core, dispensable, and private nodes in each haplotype path. Panels b–d share the same legend. Abbreviations: Hap, Haplotype.

To further evaluate sequence diversity among the *PdR1* haplotypes, we constructed a sequence graph including both *PdR1* and *PdR1^−^* haplotypes. This graph comprised 92,807 nodes spanning 1,442,276 bp. An extensive and complex bubble located approximately three-quarters along the graph indicated divergent paths consistent with large structural variation in this region (Fig. 2a). Several smaller bubbles elsewhere in the graph reflected finer-scale polymorphisms, indicating substantial structural divergence concentrated in a specific region of the locus, together with additional localized variation among haplotypes.

To characterize this variation, we classified graph nodes into three categories: core nodes (shared across all seven haplotypes), dispensable nodes (present in two to six haplotypes), and private nodes (specific to a single haplotype) (Supplementary Fig. 6). Nearly half of the nodes were dispensable (49.1%), whereas core and private nodes accounted for 29.0 and 21.9%, respectively (Fig. 2b). Private nodes were most abundant in the *V. vinifera PdR1*^−^ haplotype (10,711 nodes), and, after core nodes, represented the largest contribution to node content, followed by nodes shared among *V. arizonica* and its hybrids (Supplementary Fig. 4). This pattern indicates marked sequence divergence between *V. vinifera* and *V. arizonica*–derived haplotypes in this region. Among *PdR1* haplotypes, *PdR1c* contained the greatest number of private nodes (2,334), followed by *PdR1e* (1,253), and the two haplotypes of b43-17 (476 and 550). Although fewer than dispensable nodes, private nodes accounted for a greater proportion of total graph length (37% vs 22.2%) (Fig. 2c and d), indicating that private nodes tend to be longer than shared nodes.

**Fig. 3.**
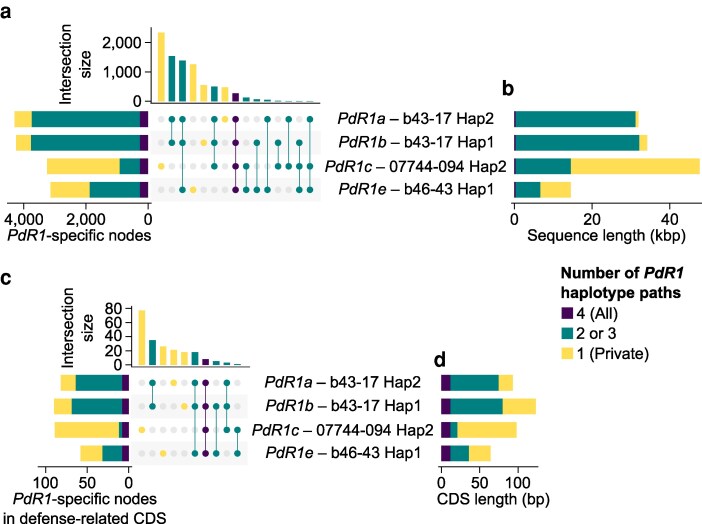
Nodes specific to the paths of the *PdR1* haplotypes in the sequence graph. a) Number of *PdR1*-specific nodes (i.e. found only in the paths of the *PdR1* haplotypes) in each *PdR1* haplotype path, and their distribution among the paths. b) Cumulative sequence length of the *PdR1*-specific nodes in each haplotype path. Number of *PdR1*-specific nodes overlapping the protein-coding sequence (CDS) of the genes potentially involved in biotic stress response, their repartition among the paths (c), and their cumulative length (d). Panels a–d share the same legend. Abbreviations: Hap, Haplotype.

Comparison of *PdR1* and *PdR1^−^* haplotype paths identified 8,562 *PdR1*-specific nodes, defined as nodes present in at least one *PdR1* haplotype and absent from all *PdR1^−^* haplotypes, representing 9.2% of all graph nodes. All four *PdR1* haplotype paths contained *PdR1*-specific nodes (Fig. 3a), ranging from 3,139 in *PdR1e* to 4,295 in *PdR1b*. The two b43-17 haplotypes contained the highest numbers of *PdR1*-specific nodes (3,479 and 3,505 in *PdR1a* and *PdR1b*, respectively). Most *PdR1*-specific nodes were shared among multiple haplotype paths, particularly between the two b43-17 haplotypes and *PdR1e*. In contrast, *PdR1c* exhibited a distinct pattern, with 71.8% of its *PdR1*-specific nodes being private, accounting for the largest cumulative private-node length (33.1 kbp) (Fig. 3b).

Among *PdR1*-specific nodes, 212 overlapped protein-coding sequences (CDSs) of defense-related genes, corresponding to 2.5% of all *PdR1*-specific nodes. These nodes were detected across all four *PdR1* haplotype paths, with the highest numbers in *PdR1b* and *PdR1c* (90 and 89, respectively), followed by *PdR1a* (82) and *PdR1e* (58) (Fig. 3c). Overall, these nodes overlapped approximately half of the defense-related gene content of each *PdR1* haplotype, ranging from 52.4 to 53.7%. They affected all defense-related gene categories, albeit in different proportions among haplotype paths (Supplementary Table 19). Only eight nodes in defense-related CDSs were shared across all four *PdR1* haplotypes, six of which overlapped genes involved in cell wall modification (Fig. 3c, Supplementary Table 19).

Most *PdR1*-specific, defense-related nodes were private to *PdR1c* (86.5%), whereas nodes in the two b43-17 haplotypes were largely shared between each other and with *PdR1e*. In *PdR1c*, private nodes predominantly overlapped CDSs of LRR-RLP genes (45 nodes) and genes involved in ROS production (16 nodes) (Supplementary Table 19). *PdR1a* and *PdR1b* shared nodes overlapping genes encoding extracellular receptors (LRR-RLPs (14) and LRR-RLK (2)), the metal–nicotianamine transporter YSL6-like protein (9), and cell wall modification genes (7). In contrast, *PdR1e* shared 24 *PdR1*-specific, defense-related nodes with one or two other *PdR1* haplotypes, mostly with the b43-17 haplotypes, and contained 26 private nodes, which overlapped genes associated with cell death and genes with putative roles in biotic stress responses of unknown mechanism.

In summary, the sequence graph analysis revealed that each *PdR1* haplotype path contains *PdR1*-specific nodes, but with distinct distributions, lengths, and functional enrichments, affecting different categories of defense-related genes.

### *PdR1c* candidate genes

**Fig. 4.**
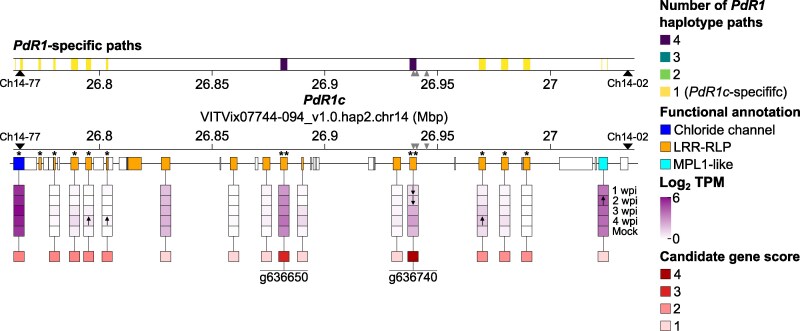
Narrowing candidate *PdR1c* genes using sequence graph information and gene expression. *PdR1*-specific paths in the defense-related genes of the refined *PdR1c* region. Gene content of the *PdR1c* locus with the defense-related genes colored according to their functional annotation. Genes with a protein sequence specific to *PdR1c* are indicated with *, genes with a protein sequence specific to all *PdR1* haplotypes are depicted with **. Expression of the defense-related genes in stems of 3 PD-resistant 07744 lines at 1, 2, 3, 4 weeks post *Xf* inoculation (wpi) and stems mock-inoculated (Mock). Gene expression is represented as the log_2_-transformed mean Transcripts per Million (TPM) for the genes expressed in at least one condition (TPM > 0). Genes more highly and lowly expressed are indicated by ↑ and ↓, respectively. Score of the defense-related genes of the refined *PdR1c* region as candidate genes. Scoring was made based on the position of the genes regarding the PD resistance-associated kmers, the specificity of their protein sequence to all *PdR1* haplotypes and *PdR1c*, and gene expression. Black triangles represent the position of the *PdR1*-associated genetic markers. Gray triangles indicate the positions of the PD resistance–associated kmers.

Because the assembly of 07744-094 (*PdR1c^+^/PdR1^−^*) enabled unambiguous phasing of *PdR1c* and the sequence graph revealed *PdR1*–specific features of the region, we investigated the *PdR1c* gene content to identify candidate genes associated with PD resistance. We previously refined the boundaries of the *PdR1* region in *V. arizonica* b40-14 between the markers Ch14-77 and Ch14-02 (Morales-Cruz et al. 2023). In *PdR1c*, these markers delimited a region spanning 26,764,799–27,034,556 bp on chromosome 14 of Haplotype 2 of 07744-094. This refined region encompassed 39 genes, including 18 defense-related genes encoding a chloride channel protein, 16 LRR-RLPs, and an MPL1-like protein (Fig. 4, Supplementary Table 14). Sequence graph analysis identified 709 *PdR1*-specific nodes within this interval, 97% of which were private to *PdR1c* (Supplementary Table 20).

To assess the functional impact of *PdR1*-specific variation, we compared CDS paths of defense-related genes in *PdR1c* with those of the three other *PdR1* and three *PdR1^−^* haplotypes (Fig. 4). *PdR1*-specific CDS paths were detected in 12 defense-related genes, encoding the chloride channel protein, 10 LRR-RLPs, and the MPL1-like protein. All *PdR1*-specific CDS paths were unique to *PdR1c*, except for two monoexonic LRR-RLP genes (g636650 and g636740) (Supplementary Table 21). Comparison of CDS paths and predicted protein sequences showed that nine genes encoded *PdR1c*-specific proteins, including the chloride channel protein gene and eight LRR-RLP genes, whereas g636650 and g636740 encoded identical protein sequences across all *PdR1* haplotypes. Notably, g636740 overlapped PD resistance–associated kmers, previously identified using 167 *V. arizonica* accessions and hybrids, providing independent support for its association with PD resistance (Morales-Cruz et al. 2023). Forty-six kmers aligned perfectly (Supplementary Table 22), spanning 26,939,281–26,945,206 bp within the Ch14-77–Ch14-02 interval, which contains only the CDS of g636740.

To further narrow down candidate genes, we profiled the expression of defense-related genes in *Xf-*inoculated stems from three PD-resistant 07744 backcross lines, including 07744-094, at 1, 2, 3, and 4 weeks post-inoculation, as well as in mock-inoculated controls. Sixteen of the 18 defense-related genes were expressed (TPM > 0) in at least one condition (Fig. 4). These included the chloride channel protein, 14 LRR-RLPs, and the MPL1-like gene. Only four genes showed differential expression in response to *Xf*, comprising four LRR-RLP genes and the MPL1-like gene.

Based on these results, we scored defense-related genes within the refined *PdR1c* region as candidate PD resistance genes using three criteria: (i) proximity to PD resistance–associated kmers, (ii) protein sequence specificity to all *PdR1* haplotypes and/or *PdR1c*, and (iii) constitutive expression and/or transcriptional response to *Xf* infection (Fig. 4; Supplementary Table 23). The LRR-RLP gene g636740 had the highest score (4), followed by the LRR-RLP gene g636650 (3). Eight genes encoding the chloride channel protein and 7 LRR-RLPs had a score of 2, while 6 genes coding for the MPL1-like and 5 LRR-RLPs had a score of 1. Accordingly, LRR-RLP genes g636740 and g636650 are the strongest *PdR1c* candidates.

## Discussion

Based on bacterial titer after inoculation with *Xf*, *V. arizonica* b40-14 have similar level of PD resistance to the two *V. arizonica* hybrids b43-17 and b46-43 (Huerta-Acosta et al. 2023; Morales-Cruz et al. 2023). However, the presence of multiple marker-defined haplotypes at *PdR1* in *V. arizonica* and its hybrids (*PdR1a-f*) suggests that PD resistance may involve different genes or allelic variants within the locus. To answer this hypothesis, we evaluated the sequence diversity among the five *PdR1* haplotypes (*PdR1a,b,c,e,f*) by first assembling and haplotyping the diploid genome of b43-17 (*PdR1a*^+^*/PdR1b*^+^), the backcross 07744-094 (*PdR1c*^+^/*PdR1*^−^), and b46-43 (*PdR1e*^+^*/PdR1f*^+^), and an accession which PD resistance is not associated with *PdR1*, b42-26 (*PdR1*^−^/*PdR1*^−^) (Table 1) (Riaz et al. 2018). Genome assembly using HiFi reads allowed to generate all *PdR1* and *PdR1*^−^ haplotypes in a single contig, which is an improvement for *PdR1c* compared to two fragmented loci in the genome assembly of *V. arizonica* b40-14 (*PdR1c*^+^*/PdR1d*^+^) (Supplementary Fig. 1). Localization of the *PdR1* region through the *in silico* amplification of *PdR1*-associated genetic markers showed differences in amplicon size and locus length between accessions and within each accession, except in b46-43, where both haplotypes were identical, with the exception of 1 SNP. Differences were also found in the gene and repeat contents of the *PdR1* haplotypes, especially in the number of LRR-RLP genes. All these differences provide clear evidence of sequence diversity between *PdR1* and *PdR1*^−^ haplotypes, as well as among the *PdR1* haplotypes themselves.

Sequence diversity was further investigated through the construction of a sequence graph including 7 haplotypes. These consisted of 4 *PdR1* haplotypes: *PdR1a*, *PdR1b*, *PdR1c*, and *PdR1e*, and 3 *PdR1*^−^ haplotypes: one from *V. vinifera* and two from the PD-resistant accession b42-26. The 2D representation of the sequence graph (Fig. 2a) showed an extensive structural divergence located approximately three-quarters along the graph, together with additional localized structural variations among haplotypes. Categorization of the graph nodes as core, dispensable, and private determined a substantial sequence divergence between *V. vinifera* and *V. arizonica*–derived haplotypes in the locus (Fig. 2b–d). Interspecific sequence diversity has also been observed in other grape disease resistance loci, such as the powdery mildew-resistance loci *Run1.2* and *Run2.2* (Cochetel et al. 2021), *Ren6* and *Ren7* (Massonnet et al. 2025), and the downy mildew-resistance loci *Rpv3* (Wilkerson et al. 2025). Using the inter- and intra-specific sequence diversity between the 4 *PdR1* haplotypes and the *V. vinifera PdR1*^−^ haplotype of 07744-094 and the two haplotypes of b42-26, we determined the nodes specific to the *PdR1* haplotypes (i.e. found in at least one *PdR1* haplotype but absent in all *PdR1*^−^ haplotypes). Investigating the *PdR1*-specific nodes showed a diversity in sequence between the four *PdR1* haplotypes (Fig. 3), while examining their overlap with defense-related genes showed that they affected different defense-related gene categories. *PdR1a* and *PdR1b* were found to share most of their *PdR1*-specific features, whereas *PdR1c* contained the highest number of private nodes, followed by *PdR1e*. Normalized median DNA-seq coverage and location of small polymorphisms in the five *PdR1b*^+^ cultivars compared to b43-17's *PdR1a* and *PdR1b* indicated a high level of homozygosity between the two haplotypes for nearly three-quarters of the locus (Fig. 1b). This corroborates the great number of *PdR1*-specific features shared between *PdR1a* and *PdR1b*. On the other hand, *PdR1a* and *PdR1b* also shared a great sequence specificity compared to *PdR1c* and *PdR1e*. Shared nodes by *PdR1a* and *PdR1b* (1,540) and their respective private nodes (476 and 550, respectively) represent a total of 2,016 and 2,090 nodes, respectively (Fig. 3a), which is slightly lower than the number of private nodes in *PdR1c* (2,334) but higher than *PdR1e* (1,253). The three *PdR1*^+^ accessions (b40-14, b43-17, and b46-43) were collected from three different collection sites in Texas and Mexico (Riaz et al. 2018), which could explain the sequence diversity among their *PdR1* haplotypes.

Finally, we demonstrated the utility of the sequence graph to narrow down candidate genes in the refined region of *PdR1c*, especially when coupled with gene expression. Scoring of the expressed defense-related genes within the refined *PdR1c* region based on proximity to PD resistance–associated kmers, protein sequence specificity, and gene expression, pointed two strong *PdR1c* candidates: the LRR-RLP genes g636740 and g636650 (Fig. 4). Because these two LRR-RLP genes are present in all the four *PdR1* haplotypes, this suggests that the same *R* gene(s) are potentially involved in PD resistance in all the three *PdR1*^+^ accessions. To answer this, it would be necessary to profile their gene expression in petioles or stems at both the constitutive level and in response to *Xf*. Monitoring the gene expression of the defense-related genes among the 3 other *PdR1* haplotypes would also help identify any additional candidate *R* gene(s). Two LRR-RLP genes were also proposed as candidate genes in *V. arizonica* b40-14 (Morales-Cruz et al. 2023). These genes correspond to g636600 and g636650 in the 07744-094 genome. Based on the results of our sequence graph, g636650 is a very strong candidate as its protein sequence is identical for all the *PdR1* haplotypes and absent in all *PdR1*^−^ haplotypes. However, the protein sequence of g636600 was also found present in b42-26, decreasing its score as a candidate gene. Gene expression profiling of *Xf*-infected tissues of b42-26 could help to determine whether g636600 is still a strong candidate. Regarding g636740, the gene was absent in the genome of *V. arizonica* b40-14. In the b40-14 genome, the refined *PdR1* region was made of 2 and 4 contigs on Haplotype 1 and 2, respectively (Morales-Cruz et al. 2023)(Supplementary Fig. 1). The fragmentation of the locus region and the absence of haplotyping information for the genome assembly are likely at the origin of the absence of g636740 in the genome of *V. arizonica* b40-14.

Future work will focus on the functional validation of the *PdR1c* candidate genes, the incorporation of additional *PdR1* haplotypes in the sequence graph, and the profiling of the gene expression of the defense-related genes in several *PdR1* haplotypes, at both the constitutive level and in response to *Xf*. This would help to determine whether the PD resistance associated with the four *PdR1* haplotypes is based on the expression of the same gene(s). As a protocol for stable protoplast-based gene editing has been developed for *V. arizonica* (Tricoli and Debernardi 2024), stable gene editing of the *PdR1c* candidate genes in *V. arizonica* b40-14 (*PdR1c*^+^*/PdR1d*^+^) could be an option. However, the lack of sequence information about *PdR1d* might complicate the process. Stable expression of the candidate genes in *V. vinifera* could be another option to assess the role of these genes in PD resistance. On the other hand, using transient transformation methods, such *as Agrobacterium*-mediated expression (Zhang et al. 2024), virus-induced gene silencing (Yang et al. 2022), and exogenous dsRNA-based RNAi (Marcianò et al. 2021), would likely be more challenging to evaluate the function of the candidate genes as *Xf* is a xylem-restricted pathogen.

From a breeding perspective, the comparison of the *PdR1* and *PdR1*^−^ haplotypes through the sequence graph could greatly help the development of precise markers, either specific to all *PdR1* haplotypes or specific to each single haplotype. Such markers could accelerate the screening in traditional breeding programs or the incorporation of the genes responsible for PD resistance in elite cultivars using gene editing (Greenwood et al. 2023).

## Data Availability

HiFi reads are accessible through NCBI under the BioProject PRJNA1380937. Genome sequences and their annotation files are available at Zenodo doi: 10.5281/zenodo.17925270. Supplemental Material available at figshare: https://doi.org/10.25387/g3.31864987.

## References

[jkag115-B1] Alam ST, et al 2022. Opposing effects of *MYZUS PERSICAE-INDUCED LIPASE 1* and jasmonic acid influence the outcome of *Arabidopsis thaliana*-fusarium graminearum interaction. Mol Plant Pathol. 23:1141–1153. 10.1111/mpp.13216. 35396792 PMC9276950

[jkag115-B2] Barnett DW, et al 2011. BamTools: a C++ API and toolkit for analyzing and managing BAM files. Bioinformatics. 27:1691–1692. 10.1093/bioinformatics/btr174. 21493652 PMC3106182

[jkag115-B3] Bolger AM, Lohse M, Usadel B. 2014. Trimmomatic: a flexible trimmer for Illumina sequence data. Bioinformatics. 30:2114–2120. 10.1093/bioinformatics/btu170. 24695404 PMC4103590

[jkag115-B4] Camacho C, et al 2009. BLAST+: architecture and applications. BMC Bioinformatics. 10:421. 10.1186/1471-2105-10-421. 20003500 PMC2803857

[jkag115-B5] Castro C, DiSalvo B, Roper MC. 2021. *Xylella fastidiosa*: a reemerging plant pathogen that threatens crops globally. PLoS Pathog. 17:. 10.1371/journal.ppat.1009813. PMC8428566 34499674

[jkag115-B6] Cheng H et al 2021. Haplotype-resolved de novo assembly using phased assembly graphs with hifiasm. Nat Methods. 18:170–175. 10.1038/s41592-020-01056-5. 33526886 PMC7961889

[jkag115-B7] Chin C-S, et al 2016. Phased diploid genome assembly with single molecule real-time sequencing. Nat Methods. 13:1050–1054. 10.1038/nmeth.4035. 27749838 PMC5503144

[jkag115-B8] Cochetel N, et al 2021. Diploid chromosome-scale assembly of the *Muscadinia rotundifolia* genome supports chromosome fusion and disease resistance gene expansion during *Vitis* and *Muscadinia* divergence. G3 (Bethesda). 11:jkab033. 10.1093/g3journal/jkab033. 33824960 PMC8049426

[jkag115-B9] Cochetel N, et al 2023. A super-pangenome of the North American wild grape species. Genome Biol. 24:290. 10.1186/s13059-023-03133-2. 38111050 PMC10729490

[jkag115-B10] Cochetel N, et al 2025. Phased epigenomics and methylation inheritance in a historical *Vitis vinifera* hybrid. Genome Biol. 26:392. 10.1186/s13059-025-03858-2. 41250217 PMC12621364

[jkag115-B11] Di Tommaso P, et al 2017. Nextflow enables reproducible computational workflows. Nat Biotechnol. 35:316–319. 10.1038/nbt.3820. 28398311

[jkag115-B12] Eddy SR . 2011. Accelerated profile HMM searches. PLoS Comput Biol. 7:e1002195. 10.1371/journal.pcbi.1002195. 22039361 PMC3197634

[jkag115-B13] El-Gebali S, et al 2019. The Pfam protein families database in 2019. Nucleic Acids Res. 47:D427–D432. 10.1093/nar/gky995. 30357350 PMC6324024

[jkag115-B14] Greenwood JR, Zhang X, Rathjen JP. 2023. Precision genome editing of crops for improved disease resistance. Curr Biol. 33:R650–R657. 10.1016/j.cub.2023.04.058. 37279695

[jkag115-B15] Guarracino A, et al 2022. ODGI: understanding pangenome graphs. Bioinformatics. 38:3319–3326. 10.1093/bioinformatics/btac308. 35552372 PMC9237687

[jkag115-B16] Haas BJ, et al 2008. Automated eukaryotic gene structure annotation using EVidenceModeler and the program to assemble spliced alignments. Genome Biol. 9:R7. 10.1186/gb-2008-9-1-r7. 18190707 PMC2395244

[jkag115-B17] Haviland DR, Stone-Smith B, Gonzalez M. 2021. Control of Pierce's disease through areawide management of glassy-winged sharpshooter (Hemiptera: Cicadellidae) and roguing of infected grapevines. J Integr Pest Manag. 12:1–10. 10.1093/jipm/pmab008.

[jkag115-B18] Heumos S, et al 2024. Cluster-efficient pangenome graph construction with nf-core/pangenome. Bioinformatics. 40:btae609. 10.1093/bioinformatics/btae609. 39400346 PMC11568064

[jkag115-B19] Huerta-Acosta KG, Riaz S, Tenscher A, Walker MA. 2023. Genetic characterization of Pierce's disease resistance in a *Vitis arizonica/monticola* wild grapevine. Am J Enol Vitic. 74:0740003. 10.5344/ajev.2022.22021.

[jkag115-B20] Jaillon O, et al 2007. The grapevine genome sequence suggests ancestral hexaploidization in major angiosperm phyla. Nature. 449:463–467. 10.1038/nature06148. 17721507

[jkag115-B21] Krivanek AF, Riaz S, Walker MA. 2006. Identification and molecular mapping of *PdR1*, a primary resistance gene to Pierce's disease in *Vitis*. Theor Appl Genet. 112:1125–1131. 10.1007/s00122-006-0214-5. 16435126

[jkag115-B22] Krogh A, Larsson B, von Heijne G, Sonnhammer ELL. 2001. Predicting transmembrane protein topology with a hidden markov model: application to complete genomes1. J Mol Biol. 305:567–580. 10.1006/jmbi.2000.4315. 11152613

[jkag115-B23] Li H, Durbin R. 2009. Fast and accurate short read alignment with burrows–wheeler transform. Bioinformatics. 25:1754–1760. 10.1093/bioinformatics/btp324. 19451168 PMC2705234

[jkag115-B24] Lomsadze A, Ter-Hovhannisyan V, Chernoff YO, Borodovsky M. 2005. Gene identification in novel eukaryotic genomes by self-training algorithm. Nucleic Acids Res. 33:6494–6506. 10.1093/nar/gki937. 16314312 PMC1298918

[jkag115-B25] Love MI, Huber W, Anders S. 2014. Moderated estimation of fold change and dispersion for RNA-seq data with DESeq2. Genome Biol. 15:550. 10.1186/s13059-014-0550-8. 25516281 PMC4302049

[jkag115-B26] Marçais G, et al 2018. MUMmer4: a fast and versatile genome alignment system. PLoS Comput Biol. 14:e1005944. 10.1371/journal.pcbi.1005944. 29373581 PMC5802927

[jkag115-B27] Marcianò D, et al 2021. RNAi of a putative grapevine susceptibility gene as a possible downy mildew control strategy. Front Plant Sci. 12:667319. 10.3389/fpls.2021.667319. 34127927 PMC8196239

[jkag115-B28] Massonnet M, et al 2025. Dissection of the *Ren6* and *Ren7* powdery mildew resistance loci in *Vitis piasezkii* DVIT2027 using phased parental–progeny genomes and intraspecific locus graph reconstruction. G3 (Bethesda). 15:jkaf250. 10.1093/g3journal/jkaf250. 41121521 PMC12693563

[jkag115-B29] Michelmore RW, Christopoulou M, Caldwell KS. 2013. Impacts of resistance gene genetics, function, and evolution on a durable future. Annu Rev Phytopathol. 51:291–319. 10.1146/annurev-phyto-082712-102334. 23682913

[jkag115-B30] Minio A, et al 2019. Iso-seq allows genome-independent transcriptome profiling of grape berry development. G3 (Bethesda). 9:755–767. 10.1534/g3.118.201008. 30642874 PMC6404599

[jkag115-B31] Minio A, et al 2022. Assembly of complete diploid-phased chromosomes from draft genome sequences. G3 (Bethesda). 12:jkac143. 10.1093/g3journal/jkac143. 35686922 PMC9339290

[jkag115-B32] Morales-Cruz A, et al 2023. Multigenic resistance to *Xylella fastidiosa* in wild grapes (*Vitis* sps.) and its implications within a changing climate. Commun Biol. 6:580. 10.1038/s42003-023-04938-4. 37253933 PMC10229667

[jkag115-B33] Patro R, et al 2017. Salmon provides fast and bias-aware quantification of transcript expression. Nat Methods. 14:417–419. 10.1038/nmeth.4197. 28263959 PMC5600148

[jkag115-B34] Quinlan AR . 2014. BEDTools: the Swiss-army tool for genome feature analysis. Curr Protoc Bioinformatics. 47:11.12.1–11.12.34. 10.1002/0471250953.bi1112s47. PMC4213956 25199790

[jkag115-B35] Rapicavoli J, et al 2018. *Xylella fastidiosa*: an examination of a re-emerging plant pathogen. Mol Plant Pathol. 19:786–800. 10.1111/mpp.12585. 28742234 PMC6637975

[jkag115-B36] Riaz S, et al 2008. Fine-scale genetic mapping of two Pierce's disease resistance loci and a major segregation distortion region on chromosome 14 of grape. Theor Appl Genet. 117:671–681. 10.1007/s00122-008-0802-7. 18516585

[jkag115-B37] Riaz S, Huerta-Acosta K, Tenscher AC, Walker MA. 2018. Genetic characterization of *Vitis* germplasm collected from the southwestern US and Mexico to expedite Pierce's disease-resistance breeding. Theor Appl Genet. 131:1589–1602. 10.1007/s00122-018-3100-z. 29713731

[jkag115-B38] Riaz S, Krivanek AF, Xu K, Walker MA. 2006. Refined mapping of the Pierce's disease resistance locus, *PdR1*, and sex on an extended genetic map of *Vitis rupestris* x *V. arizonica*. Theor Appl Genet. 113:1317–1329. 10.1007/s00122-006-0385-0. 16960717

[jkag115-B39] Shi X, et al 2023. The complete reference genome for grapevine (*Vitis vinifera* L.) genetics and breeding. Hortic Res. 10:uhad061. 10.1093/hr/uhad061. 37213686 PMC10199708

[jkag115-B40] Smit A, Hubley R, Green P. 2013. http://www.repeatmasker.org.

[jkag115-B41] Soneson C, Love MI, Robinson MD. 2016. Differential analyses for RNA-seq: transcript-level estimates improve gene-level inferences. F1000Res. 4:1521. 10.12688/f1000research.7563.2. PMC4712774 26925227

[jkag115-B42] Stanke M, et al 2006. AUGUSTUS: ab initio prediction of alternative transcripts. Nucleic Acids Res. 34:W435–W439. 10.1093/nar/gkl200. 16845043 PMC1538822

[jkag115-B43] Sun Q, Sun Y, Walker MA, Labavitch JM. 2013. Vascular occlusions in grapevines with Pierce's disease make disease symptom development Worse1[OA]. Plant Physiol. 161:1529–1541. 10.1104/pp.112.208157. 23292789 PMC3585614

[jkag115-B44] Tricoli DM, Debernardi JM. 2024. An efficient protoplast-based genome editing protocol for *Vitis* species. Hortic Res. 11:uhad266. 10.1093/hr/uhad266. 38895602 PMC11184525

[jkag115-B45] Tumber K, Alston J, Fuller K. 2014. Pierce's disease costs California $104 million per year. CalAg. 68:20–29. 10.3733/ca.v068n01p20.

[jkag115-B46] Wang M, Kong L. 2019. Pblat: a multithread blat algorithm speeding up aligning sequences to genomes. BMC Bioinformatics. 20:28. 10.1186/s12859-019-2597-8. 30646844 PMC6334396

[jkag115-B47] Waterhouse RM, et al 2018. BUSCO applications from quality assessments to gene prediction and phylogenomics. Mol Biol Evol. 35:543–548. 10.1093/molbev/msx319. 29220515 PMC5850278

[jkag115-B48] Wilkerson D, et al 2025. Comparative genomics of *Rpv3*, a multiallelic downy mildew resistance locus in grapevine (*Vitis* sp.). OENO One. 59. 10.20870/oeno-one.2025.59.1.8244.

[jkag115-B49] Wu TD, Watanabe CK. 2005. GMAP: a genomic mapping and alignment program for mRNA and EST sequences. Bioinformatics. 21:1859–1875. 10.1093/bioinformatics/bti310. 15728110

[jkag115-B50] Yang B, et al 2022. VvANR silencing promotes expression of VvANS and accumulation of anthocyanin in grape berries. Protoplasma. 259:743–753. 10.1007/s00709-021-01698-y. 34448083

[jkag115-B51] Zhang Q, et al 2024. Ectopic and transient expression of VvDIR4 gene in Arabidopsis and grapes enhances resistance to anthracnose via affecting hormone signaling pathways and lignin production. BMC Genomics. 25:895. 10.1186/s12864-024-10830-0. 39342082 PMC11439227

